# Report by the Educational Committee on Communications

**Published:** 1893-07

**Authors:** 


### Report by the Education Committee, on Communications

— FROM —

(*a*) BRITISH DENTAL ASSOCIATION;
(*b*) BALTIMORE DENTAL COLLEGE.

MEMBERS OF COMMITTEE.

DR. LEISHMAN, *Chairman.*

| | |
|---|---|
| SIR JOHN BANKS, *K.C.B.* | DR. MACALISTER, |
| DR. BRUCE, | DR. KIDD, |
| DR. GLOVER, | DR. TUKE, |
| REV. DR. HAUGHTON, | MR. WHEELHOUSE. |

The Education Committee have to report under the following reference from the Executive Committee:—

On November 21, 1892, the Executive Committee received and considered the following communications:—

(*a*) FROM THE BRITISH DENTAL ASSOCIATION.

(a) "*Memorial as to the Recognition of American Dental Diplomas in the United Kingdom.*

"We, the undersigned Licentiates of Dental Surgery of the Royal College of Surgeons, desire, respectfully, to call the attention of the Medical Council to the following facts:—

"After the passing of the *Dentists' Act* in 1878, and the settlement of the Dental Curriculum of the United Kingdom by the Medical Council, the question of the Recognition of Foreign Diplomas for Registration was raised.

"It was then, on examination, found that no foreign Dental Diploma testified so complete and so lengthened an education as the United Kingdom qualification. But it was, we believe, considered desirable on the ground of professional amenity to recognize, 'for the time being,' the Harvard and Michigan qualification, as those, though falling short, made the nearest approach to our own standard, thus waving, for a time, equality of qualification as between United Kingdom and Foreign qualifications in favor of the applicant for registration.

"The great advances which have been made from time to time in our educational standard have rendered the inequality still more marked.

"The preliminary examination demanded by these Colleges is, as may be seen from their own prospectuses, of such a nature as to fall far below the standard required from the Dental students of this country, and, further, these examinations are in some instances committed to the care of individuals who have no kind of responsibility nor any claim to be considered competent for such an office.

"On these grounds we beg, respectfully, to suggest that the Medical Council, by virtue of the power invested in it by Clause 10 of the *Dentists Act*, withdraw, for the time being, the right of registration which has hitherto been accorded to the American Colleges.

"*To the General Medical Council.*"

(The following Signatures were appended to this Memorial:)

| | |
|---|---|
| John Tomes, | Arthur S. Underwood, |
| J. S. Turner, | Joseph Walker, |
| Morton Smale, | F. Newland Pedley, |
| S. J. Hutchinson, | Francis Ewbank, |
| W. B. Paterson, | John Ackery, |
| Ashley Gibbings, | J. Howard Mummery, |
| William Hern, | Sidney Spokes, |
| Frederick Canton, | Charles S. Tomes, |

Storer Bennett.

(b) *Remarks on the English and American Dental Diplomas.*

"The most obvious difference between the American and English Dental Diplomas may be best observed in studying the methods of examination pursued in each case. Herewith are columns showing the subjects in the preliminary examination required from intending Dental students in the American Universities recognized by the English Medical Council, and also that required by the Royal College of Surgeons of England.

"It is commonly said in America amongst those Dentists who are interested in education, that, owing to independent States being able to make their own laws, and owing to the ease with which schools and colleges can be established, the competition is in a downward direction, and that schools which have tried to take a higher standard have suffered by a diminution in the number of their students. The study of the accompanying columns, and some of the conditions attached to the examinations may go far to confirm this view of the subject.

"*English Bodies.*

"1. *English Language*, including Grammar and Composition.

"2. *Latin*, including Grammar, translations from specified authors ( ), and translations of easy passages not taken from such authors.

"3. *Elements of Mathematics*, comprising (*a*) Arithmetic, including vulgar and decimal fractions; (*b*) Algebra, including simple equations; (*c*) Geometry, including the first book of Euclid, with easy questions on the subject matter of the same.

"4. *Elementary Mechanics* of Solids and Fluids, comprising the elements of Statistics, Dynamics, and Hydrostatics.

"5. *Optional.* One must be passed. Greek, French, German, Italian, or any other modern language, Logic, Botany, Zoology, Elementary Chemistry.

"This embraces the examinations of the Oxford and Cambridge Locals or the Society of Apothecaries and the College of Perceptors.

"*University of Harvard.*

"1. *English.* Write legibly and correctly an English composition of not less than 200 words. Also to write English prose from dictation.

"2. *Physics.* A competent knowledge of Physics (such as may be obtained from Balfour Stewart's 'Elements of Physics').

"3. *Elective Subjects.* One of the following must be passed:—Latin, French, German, the Elements of Algebra, or Plane Geometry.

"Examinations are conducted in writing; spelling, grammar, and construction are considered.

"A Degree in Letters, Science, or Medicine from a recognized college or scientific school is received instead of this examination.

"Examinations are held at the Dental School in North Grove Street, Boston.

"*University of Michigan.*

"1. *English.* (*a*) A grammatical and rhetorical analysis of short selections in prose and poetry. (*b*) An essay of not less than two pages (foolscap), correct in spelling, punctuation, capital letters, grammar, sentential structure and paragraphing.

"2. *Mathematics.* (*a*) Arithmetic:—Fundamental rules, Fractions (Common and Decimal), Denominate Numbers, Percentage, Proportion, Involution and Evolution, and the Metric System of Weights and Measures. (*b*) Algebra:—Fundamental rules, Fractions, Equations of the first degree containing two or more unknown quantities. (*c*) Geometry:—Plane Geometry.

"3. *Physics.* An amount represented by Avery's 'Natural Philosophy,' or Gage's 'Introduction to Physical Science.'

"4. *Botany* or *Physical Geography.* Botany:—The elements of Vegetable Anatomy and Physiology as given in Gray's 'Lessons.' Physical Geography:—Hin-

| "*English Bodies.* | "*University of Harvard.* | "*University of Michigan.* |
|---|---|---|
| | | man's 'Eclectic,' or an equivalent. "5. *Zoology.* Packard's 'Zoology,' Briefer Course. "6. *Physiology.* Martin's. 'The Human Body.' "7. *History.* Myer's 'General History,' or an equivalent, and Higginson's or Johnson's 'History of the United States.' "8. *Latin.* Jones's 'First Latin Book,' or Harkness's 'Latin Reader,' or an equivalent in any other text-book. "Examinations are held in Ann Arbor. Candidates are expected to be present at the time. To provide for those who cannot be present, other examinations will be held at such times as may be determined by the Examining Committee. "Admission examinations are also conducted at the places and by the persons named below. (Here follows a list of eleven different Examiners in as many different towns, one being a resident in London, England.) |

"The Harvard column shows a very meagre amount of requirements, and its first condition admits of a very low standard. The second condition is vague, and may also be so low as to suit the condition of almost any applicant.

"The optional subjects are very wide, and include much of what we consider essential, and for which we have definite specifications. That Latin should be optional in a previous University examination is, to say the least of it, very remarkable.

"The Michigan column makes a brave show, but, at its best, will not bear comparison with the English one, and in the method of announcement in many instances directs the attention of the candidate to the points which he will have to meet, and in 'Plane Geometry' [Section 2, (c)] there is no provision made for riders, as there is in the English section.

"All the sections seem to tie themselves down to certain elementary hand-books from which questions are to be set, and in Latin the books prescribed—one of them deservedly popular—will hardly bear comparison with CÆSAR and OVID, from which the papers are set in the English examination, besides the translations set from other authors.

"In England the examination in Arts or the preliminary examination is conducted by independent bodies who have a clearly defined standard, which must be attained by the candidate, but who have no indication of their future intentions.

"In America the certificates of certain schools are received —certificates given by these schools to their own scholars—or the candidates are examined at the colleges which are waiting to receive them as pupils (at the Dental School in Boston for Harvard, and at Ann Arbor for Michigan), or, what is more extraordinary, private individuals are empowered to examine candidates and to certify to their fitness to become students at a University which, in the course of three years at the most, may confer on them the title of Doctor and the right to be registered on the English Dentists' Register. For the Michigan University there is such an examiner appointed, and he is now resident in London. This gentleman, it may be mentioned, has recently written to one of the leading Dental journals in America that the Dental schools here are 'cheap replicas' of those in America.

"THE AMERICAN CURRICULUM.

"The American curriculum is certainly both extensive and particular, and may seem superior to the English one at the first glance owing to the amount of detail that is given. These details, however, seem to point more to the limitation rather than to the extension of what is taught; as, for instance, a course of six lectures on Neurology is specified, and then it is added: 'The anatomy of the trifacial nerve being made the subject of special study.'

"The time which is allowed for the multiplicity of subjects is three years; but a case recently before the Medical Council points to the conclusion that there is an amount of elasticity in the computation of years which enables this to be modified; for a candidate for registration had accomplished the work in such a way as to receive his degree of Doctor in a period of twenty-five months; and, from all one hears, this is not a solitary instance.

"It would be a long and more or less fruitless task to analyze one of these curricula; but if the number of subjects named can be taught in three years, then the American student and the American teacher must each be superior in method and in perceptive power to those in England. To those who do not think that this superiority exists, it seems that there is a great promise of teaching, but very small possibility of education.

"Although want of practical experience precludes a direct comparison between the curricula of the two American Universities in question and that of England, there are certain side-lights which may lead us to estimate, in some degree, their relative value.

"As against the three years American time, the English curriculum demands four years, and in many instances, where the pupil resides at a distance from an educational centre, the time virtually amounts to five years. Then there is the difference in the amount of fees charged in the American course and in the English one.

"At the Michigan School the fees are £37.14*s*, and this includes fees for parts for dissection and the Diploma fee, and all such expenses. The fees for parts are estimated at two guineas (£2. 2*s*).

"At Harvard the inclusive fees are £87. 10*s*. In this amount is included all matriculation, diploma, and demonstrator's fees; but a deposit of three guineas (£3. 3*s.*) is required to cover laboratory breakages, and also a deposit of one pound five shillings (£1. 5*s.*) for parts for dissection, the unused balance to be returned. The items to cover dissection show either a very low rate for parts, or a very small amount of dissections.

"In England the dissections are defrayed by the student as he goes along, and are kept as one of the records of his work in some schools; beyond this the fees, and diploma fee, amount to £122. The expenses are thus shown to be much heavier here than in America.

"The Professional examination in England is open to Fellows of the College and to all Teachers, and is conducted by a body of examiners who are not engaged in teaching. In America the certificates which procure the degree of Doctor are granted by the teaching staff of the school, or the 'Faculty.'

"This constitutes a very marked contrast between the two systems.

"The requirements of the Medical Council from the Dental student are in every way as strict as they are from the general student of Medicine; but the *Dental Register* admits foreign diplomas from two universities as qualifications for registration, while no such privilege is awarded to any foreign diploma by the *Medical Register.*

"In the future the Dental curriculum is not likely to be made less exacting; indeed, the tendency is in the opposite direction, and it may now be taken as certain that the preliminary examination will be very much extended, and in this extension the Dental student will have to participate, so that the discrepancy between the American and English Dental student will become greater than ever; and while this obviously unfair condition exists it will be impossible for those interested in Dental education to make that advance which they so much desire."

(*b*) FROM THE BALTIMORE COLLEGE OF DENTAL SURGERY.

"BALTIMORE, JUNE 1, 1892.

"*Dear Sir:*—The Faculty of the Baltimore College of Dental Surgery most respectfully make application to have this school recognized by the Medical Council of England, and placed on the same footing with the Dental Departments of Harvard and Michigan Universities in the United States of America.

"In making this application we would state that this College is the oldest Dental institution in the world, and that the Dental profession dates its origin from the founding of this school, the Charter of which was granted by the Maryland Legislature in 1839.

"Without making invidious comparisons, we claim to be the peer and equal of any Dental school in the world—that we have always maintained a foremost position in advancing the interests and welfare of the profession as an educational institution—that our curriculum and instructions are fully equal to the demands of the times—that we probably number among our *alumni* more distinguished scientific men than any other school—and that our standing at home is unquestioned. In consideration of these facts we think it is hardly fair to discriminate against us; and, whether judged by our work already done, or by the work we are now doing, we think we are fully entitled to your consideration and recognition. Our diplomas, in this country, are recognized wherever the degrees of Harvard and Michigan are.

"We mail you our catalogue, which contains a list of all our Graduates, and will be pleased to give any further information, should it be desired.

"Very respectfully, your obedient servant,

"R. B. WINDER,

"*Dean.*

"W. J. C. MILLER, Esq., B.A.,

"*Registrar of the General Medical Council.*"

---

*** *From a printed announcement supplied by the College, it appears that the course is divided into a Freshman, Junior and Senior term.*

The studies assigned to the Freshman term comprise Anatomy, Physiology, Chemistry, Dental Materia Medica, Didactic and Practical Mechanical Dentistry and Operative Dentistry, and Pathology and Therapeutics.

The course of studies for the Junior term comprises a review of the Freshman year studies, Histology, Pathology, Materia

Medica, Therapeutics, Mechanical and Operative Dentistry, Dissection, Chemistry, Physiology, General and Surgical Anatomy.

In the Senior year the preceding studies will be continued with the addition of Surgery.

---

In regard to the foregoing communications the Executive Committee passed the following *Resolution:*

*Resolved:*—"That these communications be referred to the Education Committee for consideration, and that they be requested to report to the next session of the General Council."

On reference to the Report by the Executive Committee on Qualifications for registration under the "Dentists' Act" (1878) (Minutes, Vol. XVI, pp. 148–198), it will be found that "the (Executive) Committee were of the opinion that the certificates of the degree of Doctor of Dental Medicine granted by Harvard University, and of the Degree of Doctor of Dental Surgery granted by the University of Michigan, may be recognized by the Council, under clause 10 of the 'Dentists' Act.'" The grounds for this recommendation are as follows:—"In each of these institutions the candidate for the degree is required to have devoted three years to professional study, and to have attended lectures and courses of instruction at a Dental College during two years. The examinations, which are written and practical, including actual operations and the preparation of specimens of mechanical dentistry, appear to furnish sufficient guarantees of the possession of the requisite knowledge and skill for the efficient practice of Dentistry or Dental surgery." At this period no preliminary examination was demanded at Harvard.

The common requirements of the four British and Irish Licensing Bodies at that time (1879) were, as stated by the Executive Committee:—"In each instance the candidate is required to be twenty-one years of age, to have passed one of the preliminary examinations recognized by the Medical Council in the case of Medical Students, to have been engaged four years in the acquirement of professional knowledge, to have been engaged three years in the acquirement of practical knowledge under a registered practitioner, and to produce evidence as to moral character." Beyond these conditions, attendance on courses of Anatomy, Physiology, Surgery, Medicine, Chemistry, and Materia Medica was obligatory; the length of time for the study of each subject varying in the requirements of the different bodies.

Communications have on various occasions been received from American Universities and Colleges, begging that their licences in Dental Surgery should be recognized by the Council (Minutes, Vol. XVI. p. 438; Vol. XXVIII, pp. 214–216). These communications showed that the requirements of the petitioners had been raised to the standard of the two American Colleges recognized by the Council. In every case, however, the petition was refused on the ground that preliminary examinations were not enjoined, and that the course of professional instruction extended over three years only.

There is reason to believe that certain of the American bodies have brought themselves up to the standard of Michigan and Harvard, and that their teaching appliances are satisfactory. From personal observation the Committee have been assured of the excellence of the teaching arrangements in such centres as Baltimore and Philadelphia. It appears, therefore, to the Committee that injustice is done to the American Bodies whose curriculum is, to say the least, equal to those of Harvard and Michigan, by the grant of special recognition to the two last-named colleges alone.

It appears, however, to the Committee, that a greater injustice is done to the Licentiates of the British Bodies, by placing them on the same level as the Diplomates of the two recognized American Colleges whose curricula are manifestly inferior to those enjoined in this country. The Council can assure itself of the character of the teaching and examination of the home Licensing Bodies; and, being possessed, by Sec. 22 of the "Dentists' Act, of full power of visitation and inspection, can exercise discipline in order to maintain or raise the standard. The Council has no such power over Foreign Colleges. So far as the interests of the public are concerned, the question involved is not at present of great importance; for on reference to the "Table showing the numbers and qualifications, with percentage of the total, of persons registered in the *Dentists' Register* for 1892," it will be found that the names of only 19 Foreign Dentists appear (7 from Harvard and 12 from Michigan), forming 0·38 per cent. of all the registered Dental Practitioners. (*Dentists' Register*, 1892, Table on p. 23.)

---

### APPENDIX TO REPORT.

The following communications in regard to the Dental Degrees of the Universities of Harvard and Michigan have been referred to the Education Committee by the Executive Committee:—

(a) *Letter presented by Mr. Charles Tomes, containing Resolutions passed by London and Counties Medical Protection Society.*

"13 ROYAL AVENUE, SLOANE SQUARE,
"LONDON, S.W., March 10, 1893.

"*Dear Sir:*—I beg to inform you that at a meeting of the Council of the London and Counties Medical Protection Society on February 28th last it was unanimously *resolved:*—

"(1) That, from the facts which have come to the knowledge of the Council of this Society, it is of opinion that any recognition by the General Medical Council of the diplomas of Harvard and Michigan leads to serious injustice, and tends to lower the status of the Dental Profession in England.

"(2) That Mr. Tomes be requested to bring the above resolution to the notice of the General Medical Council.

"I am, dear Sir,
"Yours faithfully,
"HUGH WOODS,
"*Hon. Sec. L. & C. Med. Prot. Soc.*

"*C. S. Tomes, Esq., F.R.S.*"

---

(b) *Letter from Mr. Morton Smale.*

"22A, CAVENDISH SQUARE, W.
"May 18, 1893.

"*Dear Sir:*—I should like to call your attention, and through you that of the General Medical Council, to the complaint of Mr. Hill, that a German fellow-student who passed the Dental examination at Harvard at the same time with himself has been allowed a place on the *Dentists' Register*, while he (Hill) has been refused registration.

"When the two American colleges were admitted to our home *Register*, it was certainly never contemplated that they were to be able to send to us foreigners of any or all nationalities. Yet in so doing they are acting within the rights conceded to them.

"According to this, the two American colleges, having access to our *Register*, have the whole population of the world as their possible students, and, with their diplomas, are eligible for our home *Register*.

"Excepting always natives of Great Britain, therefore, these two American Colleges, with a shorter and inferior curriculum, and inferior examination, are very likely to send more foreign applicants for registration than are likely to come from our home schools.

"We consider this is an additional reason for granting the concession which has been applied for, regarding the registration of all foreign Dental diplomas.

"Yours most truly,

"MORTON SMALE.

"*W. J. C. Miller, Esq.*"

---

"No. 4808.

"GENERAL MEDICAL COUNCIL OFFICE,

"299 OXFORD STREET, LONDON, *W.*, May 31, 1893.

"*Sir*:—By order of the General Medical Council, I send you herewith a copy of a Report presented to the Council by its Education Committee, whereon the Council, at its meeting on the 29th inst. passed the following resolution:—

"That the recognition of the certificates of the degrees of Doctor of Dental Medicine of the University of Harvard, and Doctor of Dental Surgery of the University of Michigan by the General Council be suspended until further notice, and that the Registrar be instructed to refuse registration of such certificates.

"I am, Sir,

"Yours faithfully,

"W. J. C. MILLER,

"*Registrar.*

"*The Secretary of the Dental Department of the University of Michigan.*"

